# Pharmacist Awareness, Knowledge, and Clinical Engagement of Medication-Related Osteonecrosis of the Jaw: A Survey From a Designated Community Cancer Care Hospital

**DOI:** 10.7759/cureus.91892

**Published:** 2025-09-09

**Authors:** Motohiko Sano, Yosuke Iijima, Saya Watanabe, Atsunobu Sagara, Kenta Ushikubo, Miki Yamada, Hiroyuki Ohya, Mashu Morimoto, Norio Horie, Takahiro Kaneko

**Affiliations:** 1 Laboratory of Clinical Pharmacy Assessment, Hoshi University, Tokyo, JPN; 2 Department of Oral and Maxillofacial Surgery, Saitama Medical University, Saitama, JPN; 3 Department of Pharmacy, Japanese Red Cross Medical Center, Tokyo, JPN; 4 Department of Pharmacy Services, Saitama Medical University, Saitama, JPN

**Keywords:** bone-modifying agents, cancer, interprofessional collaboration, medication-related osteonecrosis of the jaw, pharmacists, questionnaire

## Abstract

Introduction

Medication-related osteonecrosis of the jaw (MRONJ) is a clinically challenging condition; however, the actual practice and interprofessional engagement of pharmacists regarding MRONJ remain undetermined. We assessed the awareness, knowledge, and clinical engagement regarding MRONJ among pharmacists working at a cancer care hospital, identified the gaps between theoretical understanding and actual clinical practice, and explored strategies for enhancing interprofessional collaboration and educational support.

Methods

In this cross-sectional survey, we administered a web-based anonymous questionnaire to 65 pharmacists working at the hospital between February 19 and March 18, 2024. The questionnaire covered topics including pharmacist demographics, clinical experience with bone-modifying agents (BMAs), understanding of MRONJ, and experiences with interprofessional collaborations in dental care.

Results

Response rate was 78.5% (51/65). Most respondents were in their 20s or 30s (33.3% for both age groups) and had <10 years of professional experience. Approximately 90% were aware of MRONJ; however, only 3.9% had encountered an actual case. Many pharmacists demonstrated an understanding of the link between BMAs and MRONJ; however, only 21.6% reported that they “sometimes” assessed oral conditions during medication counseling, and none reported doing so “always.” Notably, 45.1% pharmacists reported difficulties in collaborating with dentists, and overall interprofessional engagement remained limited. Most pharmacists expressed a positive attitude toward interprofessional collaborations, and 98.0% acknowledged the importance of working with dental professionals. However, none had provided oral health information to external dental clinics.

Conclusions

There is a gap between the theoretical knowledge of MRONJ among pharmacists and their clinical experience. Their limited involvement in outpatient dental care suggests the presence of structural barriers. Interprofessional collaboration and educational support systems need enhancement.

## Introduction

Medication-related osteonecrosis of the jaw (MRONJ) is a severe adverse event primarily associated with antiresorptive agents, such as bisphosphonates and denosumab, as well as certain antiangiogenic drugs. Since its first recognition in 2003 [1], MRONJ has posed considerable and persistent clinical challenges owing to its detrimental impact on patient quality of life and the complexity of its prevention and management. The pathogenesis of MRONJ is multifactorial; however, some contributing factors include the suppression of osteoclast-mediated bone remodeling, oral infections, and inflammation. MRONJ occurs in 4.9% of patients with cancer treated with denosumab [2]. Therefore, completing dental treatment before initiating bisphosphonates or denosumab and regular dental follow-up are essential preventive strategies.

In Japan, the number of patients with osteoporosis is increasing owing to the aging population. Furthermore, bone-modifying agents (BMAs) are widely used to manage skeletal-related events, including bone pain, hypercalcemia, spinal cord compression, and pathological fractures in patients with solid tumors and multiple myeloma. A recent survey revealed that 9.8% of long-term care-facility residents were being treated with bisphosphonates or denosumab for osteoporosis, with one case of MRONJ observed in a resident receiving injectable bisphosphonates [3]. Given the increasing population receiving these agents, the number of MRONJ cases is also expected to rise, highlighting the need for comprehensive, multidisciplinary prevention strategies involving dentists and medical and pharmaceutical professionals.

Several studies have reported the awareness and knowledge of MRONJ among dentists and dental students, consistently reporting insufficient understanding, particularly among general practitioners and those with limited experience [4-6]. Similarly, the lack of MRONJ knowledge has been reported among medical students [7]. Furthermore, dental hygienists working in private dental clinics in Japan showed limited awareness and clinical experience with MRONJ, underscoring the need for broader educational interventions across dental professions [8]. To address these educational gaps, researchers have proposed targeted educational programs, enhancement of interprofessional collaboration, and meticulous documentation of patient medication histories [9,10]. Additionally, the MRONJ-Quality of Life Questionnaire was developed to assess the effect of MRONJ on the daily functioning of patients [11]. Despite the central role of pharmacists in managing medications associated with MRONJ, their awareness and active participation in preventing MRONJ remain undetermined. Pharmacists, especially those in oncology settings, are frequently involved in initiating and monitoring antiresorptive therapies and are therefore in a unique position to identify patients at risk for MRONJ, provide patient education, and serve as a bridge between medical and dental care. However, data regarding pharmacist knowledge, their attitudes toward MRONJ, and the extent of their involvement in interdisciplinary preventive measures remain scarce.

This study aimed to assess the awareness, knowledge, and attitudes of pharmacists working in a designated community cancer care hospital in Japan regarding MRONJ, while also evaluating their engagement in dental-related practices with attention to potential gaps between theoretical understanding and clinical application. Furthermore, we sought to identify systemic barriers and propose strategies to enhance interprofessional collaboration and educational support systems for the early diagnosis and prevention of MRONJ.

## Materials and methods

Survey method

Saitama Medical Center, Saitama Medical University, a regional core hospital and designated cancer center, provides surgery, chemotherapy, and radiotherapy for various types of cancer. Pharmacists working in the hospital pharmacy department (n=65) were surveyed based on their awareness, knowledge, and clinical engagement related to MRONJ using an anonymous questionnaire created with Google Forms (Google LLC, California, US). Concurrently, a survey on the involvement of pharmacists in dental care was conducted. The survey period was one month, between February 19 and March 18, 2024. To enhance the response rate, a reminder email was sent two weeks after the initial distribution of the questionnaires.

Five sections of the questionnaire

The first section included three items about demographic characteristics: sex, age, and years of pharmacist experience. The second section included four items related to oral cavity checks during medication counseling and suggestions for oral care interventions to other healthcare providers. The third section contained nine items concerning experience with dispensing BMAs (bisphosphonates, zoledronic acid, and denosumab), medication counseling, and knowledge of associated adverse events. The fourth section included seven items to assess pharmacists’ knowledge of MRONJ and their experience with consultations for suspected cases. The fifth section comprised seven items addressing collaboration with other professions and community healthcare, including providing information to dental clinics outside the hospital and strategies for interprofessional collaboration between pharmacists and dentists. This questionnaire was developed based on previous literature and existing research on MRONJ. Its content validity was reviewed by an oncology pharmacist, a dentist specializing in oral surgery, and a faculty member from a school of pharmacy. A pilot test was subsequently conducted with two pharmacy students to confirm clarity, comprehensibility, and feasibility prior to distribution.

Ethical considerations

This study was approved by the Ethics Committee of the Center for Medical Science of Saitama University (Application No.: 2023-032). Data was anonymized and the study was conducted under the ethical standards of the Declaration of Helsinki (1964 and later amendments). Participants provided informed consent using the Google Forms platform.

## Results

Table 1 shows the participant demographic characteristics.

**Table 1:** Demographic characteristics and basic information regarding the study participants

First section	n	%
Sex		
Male	27	52.9
Female	24	47.1
Age (years)		
50-59	2	3.9
40-49	11	21.6
30-39	17	33.3
20-29	17	33.3
Unanswered	4	7.8
Years of experience as a pharmacist		
≥20	6	11.8
15-19	7	13.7
10-14	2	3.9
5-9	16	31.4
≤4	16	31.4
Unanswered	4	7.8

About 52.9% were male participants, whereas 47.1% were female participants. The most common age groups were 20s and 30s, each representing 33.3% of the cohort. Regarding experience, 31.4% of the pharmacists had less than or equal to four years of experience, and 31.4% had five to 10 years of experience.

Table 2 shows the frequency and circumstances of oral cavity checks conducted by pharmacists during medication counseling.

**Table 2:** Items in the questionnaire related to checking the oral cavity

Second section	n	%
Q. 1 Oral cavity checks during medication instruction		
Always	0	0
Sometimes	11	21.6
Almost never	21	41.2
Never	19	37.3
Q. 1-1 Related question: When would you check? (multiple choice)		
When there are oral mucosal disorders	22	78.6
When using medicines affecting the oral cavity	20	71.4
When oral mucosal disorders are anticipated	15	53.6
When there is oral dryness	5	17.9
When undergoing radiotherapy	4	14.3
When there is time to spare	4	14.3
When dentures or orthodontics are used	2	7.1
When intervening with terminally-ill patients	2	7.1
During team care rounds	1	3.6
When there is a complaint of any oral symptoms	1	3.6
When there are subjective symptoms	1	3.6
When intervening in elderly patients (over 65 years)	0	0
Q. 2 Have you ever provided medication counseling to cancer patients or their families?		
Yes	46	90.2
No	5	9.8
Q. 3 Have you ever suggested oral care to the patient's physician or dentist before or after surgery or the start of anti-cancer drug treatment?		
Yes	15	29.4
No	36	70.6

Although no pharmacists reported always checking the oral cavity, 21.6% sometimes checked, 41.2% rarely checked, and 37.3% never checked it. The most common reasons for conducting oral checks included the presence of oral mucosal damage, medication use affecting the oral cavity, and anticipation of oral mucosal damage.

Table 3 shows the pharmacists' experience with dispensing bisphosphonates and denosumab, providing medication counseling, and their knowledge of associated adverse events.

**Table 3:** Items in the questionnaire related to experience in dispensing bisphosphonates and denosumab, providing medication guidance, and knowledge of adverse events MRONJ, medication-related osteonecrosis of the jaw; ZOA, zoledronic acid.

Third section	n	%
Q. 4 Have you ever dispensed bisphosphonates?		
Yes	50	98.0
No	1	2.0
Q. 5 Have you ever dispensed denosumab?		
Yes	48	94.1
No	3	5.9
Q. 6 Have you ever given medication counseling for bisphosphonates (oral medicines)?		
Yes	45	88.2
No	6	11.8
Q. 7 Have you ever given medication counseling for bisphosphonates (injectable medicines)?		
Yes	18	35.3
No	33	64.7
Q. 8 Have you ever given denosumab medication counseling?		
Yes	18	35.3
No	33	64.7
Q. 9 Indicate known bisphosphonates and denosumab adverse events (multiple choice)		
Jawbone disorders (osteonecrosis of the jaw, jaw osteomyelitis)	50	98.0
Hypocalcemia	46	90.2
Gastrointestinal symptoms (stomatitis, oral herpes, upper abdominal pain, etc.)	27	52.9
Skin symptoms (eczema, cellulitis, etc.)	12	23.5
Back pain	9	17.6
Fatigue	8	15.7
Constipation	8	15.7
Anemia	6	11.8
Dyspnea	2	3.9
I have never heard of any of these	0	0.0
Q. 10 Have you heard that ZOA can cause MRONJ of the jaw?		
Yes	49	96.1
No	2	3.9
Q. 11 Have you heard that denosumab can cause MRONJ?		
Yes	45	88.2
No	6	11.8
Q. 12 Do you think there is a difference in the incidences of MRONJ between ZOA and denosumab?		
ZOA < denosumab	5	9.8
ZOA > denosumab	8	15.7
Similar level	9	17.6
I am not sure	29	56.9

Nearly all pharmacists had dispensed bisphosphonates (98.0%) and denosumab (94.1%). MRONJ was the most widely recognized adverse event, followed by hypocalcemia and gastrointestinal symptoms.

Table 4 shows a summary of the pharmacists' knowledge about MRONJ.

**Table 4:** Items in the questionnaire related to MRONJ knowledge and consultation experience MRONJ, medication-related osteonecrosis of the jaw.

Fourth section	n	%
Q. 13 Selected conditions diagnosed as MRONJ (multiple choice)		
Prior treatment with bisphosphonates or denosumab	31	60.8
Bone exposure in the oral, maxillofacial, or mandibular region persisting for >8 weeks	20	39.2
Prior combination of bisphosphonates or denosumab with angiogenesis inhibitors or immunomodulators	19	37.3
Not primary cancer or cancer metastasis to the jawbone	16	31.4
Exposed bone that can be probed through an intraoral or extraoral fistula in the maxillofacial region that has persisted for >8 weeks	12	23.5
In principle, no history of radiation therapy to the jawbone	12	23.5
I am not sure	18	35.3
Q. 14 Select all risk factors for developing MRONJ (multiple choice)		
Medication	45	88.2
Periodontal disease	44	86.3
Tooth extraction	39	76.5
Dental caries	37	72.5
Poor oral hygiene	36	70.6
Dental implant	32	62.7
Radiation	31	60.8
Diabetes	30	58.8
Smoking	29	56.9
Rheumatoid arthritis	24	47.1
Obesity	18	35.3
Dialysis	18	35.3
Alcohol consumption	16	31.4
I am not sure	3	5.9
Q. 15 Have you seen intraoral photographs of MRONJ?		
Yes	30	58.8
No	21	41.2
Q. 16 Have you seen actual intraoral views of MRONJ?		
Yes	2	3.9
No	49	96.1
Q. 17 Have you ever discussed a patient with suspected MRONJ with their physician or dentist?		
Yes	7	13.7
No	44	86.3
Q. 18 Selected as the highest priority treatment for MRONJ		
Surgical removal of sequestrum	24	47.1
Oral rinses	18	35.3
Antibiotics	1	2.0
Analgesics	1	2.0
I am not sure	7	13.7
Q. 19 Do you think MRONJ is a disease that can be treated and cured?		
Curable	30	58.8
Not curable	21	41.2

When asked about diagnostic conditions, the most commonly recognized criterion was prior treatment with bisphosphonates or denosumab (60.8%), followed by the presence of exposed bone in the maxillofacial region persisting for more than eight weeks (39.2%), and prior combination therapy with bisphosphonates or denosumab and angiogenesis inhibitors or immunomodulators (37.3%). Regarding the risk factors for MRONJ, medication use was most frequently identified (88.2%), whereas dental-related factors, including periodontal disease (86.3%), tooth extraction (76.5%), and dental caries (72.5%), were also highly recognized. Poor oral hygiene was cited by 70.6% of the respondents. Regarding clinical experience, 58.8% of the pharmacists had observed intraoral photographs of patients with MRONJ; however, only 3.9% had directly observed MRONJ lesions in the oral cavity. Furthermore, 86.3% had no experience of consulting a physician or dentist regarding a suspected case of MRONJ. Concerning treatment modalities, surgical removal of sequestrum was the most frequently selected approach (47.1%), followed by oral rinses (35.3%) and antibiotics (2.0%). Regarding the curability of MRONJ, 58.8% of the pharmacists believed that it could be cured, whereas 41.2% believed that it could not.

Table 5 shows the pharmacists' experiences concerning collaboration with other healthcare professions.

**Table 5:** Items in the questionnaire regarding collaboration with other professions and the medical community

Fifth section	n	%		
Q. 20 Have you ever provided information about bisphosphonates and denosumab to external medical institutions using a medication handbook or other means?				
Yes	12	23.5		
No	39	76.5		
Q. 21 Have you ever shared information about dental and oral health issues with dental clinics outside the hospital?				
Yes	0	0		
No	51	100		
Q. 22 Do you think it is necessary to cooperate with dentists?				
Yes	50	98.0		
No	1	2.0		
Q. 23 In which areas do you think collaboration between pharmacy and dentistry should be promoted? (multiple choice)				
Perioperative oral management	42	82.4		
Oral function management	32	62.7		
Feeding and swallowing management	30	58.8		
Prevention of aspiration pneumonia	26	51.0		
Home healthcare	21	41.2		
Nursing care insurance	9	17.6		
Healthcare for people with disabilities	9	17.6		
Q. 24 As a pharmacist, please select the healthcare professions with which you find it easy or difficult to collaborate. (multiple choice)	Easy		Difficult	
	ｎ	％	ｎ	％
Dentist	10	19.6	23	45.1
Dental hygienist	8	15.7	31	60.8
Physician / Doctor	43	84.3	4	7.8
Pharmacist	45	88.2	2	3.9
Nurse	45	88.2	1	2
Registered dietitian / Dietitian	17	33.3	11	21.6
Physical therapist	5	9.8	28	54.9
Medical social worker	3	5.9	33	64.7

Overall, 23.5% of the pharmacists reported having provided information about bisphosphonates and denosumab to medical institutions outside their hospital using a medication handbook or similar means. Importantly, none of the pharmacists had provided dental or oral health information to dental clinics outside the hospital. Regarding collaboration with other healthcare professionals, pharmacists indicated that they found it easier to collaborate with nurses (88.2%), fellow pharmacists (88.2%), and physicians (84.3%) than they did with dentists (45.1%) and dental hygienists (60.8%). However, 98.0% of the pharmacists agreed on the necessity of collaborating with dentists. Regarding areas where drug-dental cooperation should be promoted, perioperative oral management was most frequently selected (82.4%), followed by oral function management (62.7%), feeding and swallowing management (58.8%), prevention of aspiration pneumonia (51.0%), home healthcare (41.2%), and nursing insurance and healthcare for individuals with disability (17.6% for both).

In the free-text responses, pharmacists emphasized the importance of sharing detailed patient information with dentists to ensure appropriate dental care, particularly in patients at risk for MRONJ. The essential information included basic medical information, current medication use (especially bisphosphonates and denosumab), medication history, and history of adverse drug reactions. Additionally, pharmacists highlighted the need for sharing the history of chemotherapy (including the types of drugs used, treatment duration, and adverse events), bisphosphonate use (particularly injectable forms), and radiotherapy, which are key determinants in evaluating the risk of jawbone necrosis. Information on the use of antiplatelet or anticoagulant agents, which may increase the risk of bleeding, was also considered necessary to be communicated to dental professionals.

## Discussion

In this study, we assessed the awareness and knowledge regarding MRONJ among pharmacists working at a designated community cancer care hospital, as well as their involvement in dental care and potential strategies to strengthen their role in preventing MRONJ. Our findings revealed a substantial gap between their theoretical understanding of MRONJ and their practical engagement in its prevention and management. Despite limited clinical engagement, most pharmacists recognized the importance of collaborating with dental professionals, indicating a generally positive attitude toward interprofessional cooperation.

A high proportion of pharmacists demonstrated awareness of MRONJ and its associated risk factors, consistent with the findings of previous studies among medical and dental professionals [4,5]. Nevertheless, only 3.9% had encountered an MRONJ case in the clinic. Moreover, the limited frequency of oral cavity assessments during medication counseling highlights the disconnection between theoretical knowledge and proactive preventive practices. The need for early identification of oral risk factors, such as mucosal disorders and periodontal disease, has been emphasized as an essential factor in preventing MRONJ [12,13]; however, our findings indicate that such preventive strategies have yet to be fully integrated into routine pharmacy practice, even in oncology-focused settings where BMAs are commonly used. Notably, 98% of the pharmacists acknowledged the importance of interprofessional collaboration with dental professionals, indicating a strong prospect for future engagement. However, despite this high level of acceptance, the actions of the pharmacists did not align with their stated attitudes because none of the respondents had ever provided oral health-related information to external dental clinics. This discrepancy between awareness and practice shows that pharmacists may face practical difficulties in translating their intentions into clinical actions. This may be attributable to factors such as uncertainty about which information should be shared, lack of institutional protocols for interprofessional communication, and insufficient confidence in discussing oral health with dental professionals. These challenges highlight the presence of systemic barriers, similar to those reported in previous interdisciplinary studies [14]. These structural challenges may include the absence of formal referral pathways, lack of reimbursement incentives for pharmacist-dentist communication, and insufficient interprofessional training opportunities. Similar structural challenges have also been previously reported among dental hygienists working in private dental clinics in Japan, suggesting the need for comprehensive and system-wide efforts to reinforce interprofessional collaboration across medical and dental care settings [8].

Given these findings, three measures are recommended to bridge the gap between the theoretical knowledge of pharmacists and their limited clinical engagement in MRONJ prevention. First, structured educational interventions should be implemented to strengthen the clinical competencies of pharmacists. Structural interventions should include training in recognizing early oral signs and symptoms of MRONJ, oral examination techniques, and effective communication strategies for risk explanation to patients and dental professionals. Previous studies have shown that such targeted training can significantly enhance the preparedness of healthcare providers in preventing MRONJ [9,10], suggesting that similar programs tailored for pharmacists could be highly beneficial.

Second, it is essential to establish institutional systems that enable pharmacists to apply this knowledge practically. Developing standardized tools, such as medication handbooks with dental risk indicators, referral forms for dental evaluation, and checklists for oral risk assessment, could facilitate interprofessional communication. Additionally, creating formalized referral pathways and regular multidisciplinary meetings involving pharmacists and dental professionals would provide critical platforms for collaboration. Integrated care models used in other fields, such as osteoporosis management, may offer useful frameworks for designing these systems [15,16]. Notably, free-text responses in our study underscored the need for clear information-sharing protocols between pharmacists and dental providers, including patient histories of medication use (especially bisphosphonates and denosumab), chemotherapy, radiotherapy, and bleeding risks.

Third, broader multi-institutional research is warranted to validate these findings across diverse clinical settings and to guide the development of national or international strategies in preventing MRONJ. This study was limited to a single community cancer care hospital; nonetheless, it is among the first to empirically examine the awareness, knowledge, and interprofessional practices of pharmacists concerning MRONJ in Japan. The results provide data that can inform future policy development, educational frameworks, and collaborative models in oncology pharmacy practice.

This study has several strengths, including addressing an underexplored area by evaluating the knowledge, attitudes, and collaboration of pharmacists regarding MRONJ, and highlighting the importance of interprofessional approaches. However, several limitations should be acknowledged. First, the single-institution design may limit the generalizability of the findings to other settings or countries. Second, the reliance on self-reported data introduces the potential for reporting bias, including social desirability bias. Third, while awareness and attitudes were assessed, actual clinical skills and engagement in MRONJ-related care were not evaluated. Fourth, as this study was conducted in a single institution with a relatively small sample of 51 pharmacists, the ability to perform more detailed subgroup analyses was restricted. Despite these limitations, it provides valuable insights and offers a foundation for future multi-institutional research and educational initiatives.

## Conclusions

This exploratory study suggests that pharmacists in a designated community cancer care hospital possess substantial theoretical knowledge of MRONJ and generally hold a positive attitude toward interprofessional collaboration with dental professionals. However, their actual clinical engagement appeared limited, indicating a potential disconnection between knowledge and practice. These findings highlight the possible value of structured educational interventions and institutional support systems to help address this gap. In addition, further multi-institutional research will be important to confirm these observations and to guide the development of standardized frameworks that enhance the role of pharmacists in preventing MRONJ.
